# Supplementary material for: HS 3D-SeboSkin Model Enables the Preclinical Exploration of Therapeutic Candidates for Hidradenitis Suppurativa/Acne Inversa
Source: Pharmaceutics. 2023 Feb 12;15(2):619. doi: 10.3390/pharmaceutics15020619 (PMC9967844; doi:10.3390/pharmaceutics15020619)
Supplement: Supplementary file 1 [file pharmaceutics-15-00619-s001.zip › pharmaceutics-2172680-supplementary.pdf]

Supplementary Figures

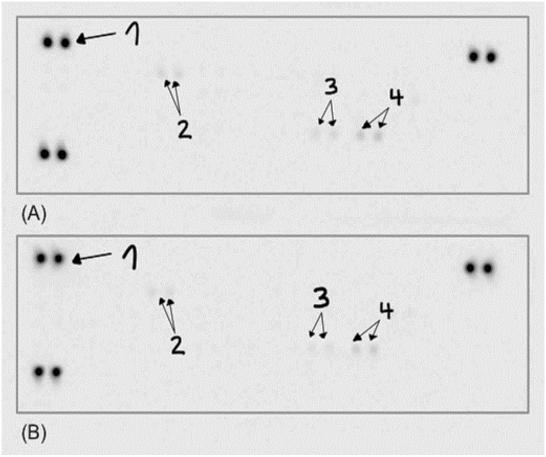

Figure S1. Representative blots of the NFκB signal pathway members TRAIL-R1/TNFRSF10A, TRAIL-R2/TNFRSF10B and CD40/TNFRSF5 in normal human NHK-19 keratinocytes after 24-h pre-incubation with linoleic acid (LA, 10^{-4} M) and subsequent treatment with LA or LA and adalimumab (ADA, 30 μg/ml) over 3 days. (A) Blotted protein membranes of the keratinocytes treated with LA. (B) Blotted protein membranes of the keratinocytes treated with LA and ADA. Reference spot; 2, CD40/TNFRSF5; 3, TRAIL-R1/TNFRSF10A; 4, TRAIL-R2/TNFRSF10B.

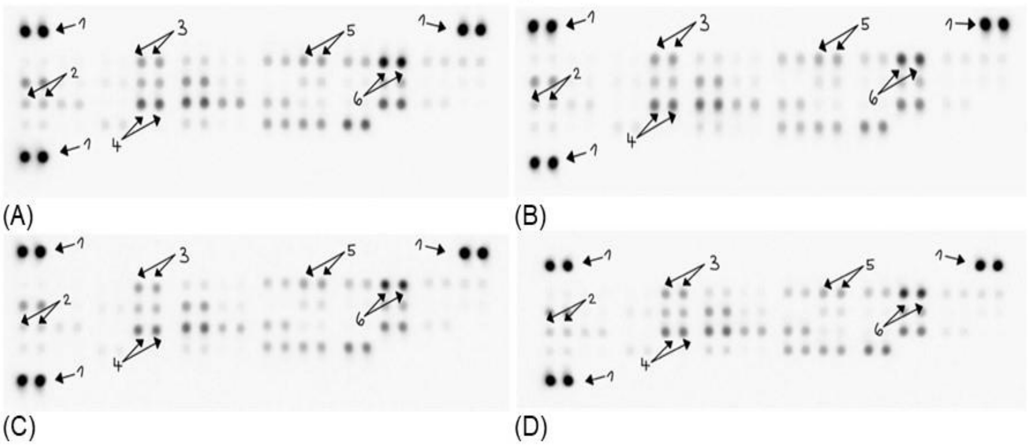

Figure S2. Representative blots of the NFκB signal pathway members NFkB1, CD40/ TNFRSF5, p53, Fas/TNFRSF6/CD95 and IκBε in human SZ95 sebocytes after 24 hour pre-incubation with linoleic acid (LA, 10^{-4} M) and subsequent treatment with LA, adalimumab (ADA, 30 μg/ml) or LA and ADA over 3 days. (A) Blotted protein membranes of the SZ95 sebocytes untreated control. (B-D) Blotted protein membranes of SZ95 sebocytes treated for 24 h with ADA (B), LA (C), and LA and ADA (D). Reference spot; 2, NFκB1; 3, CD40/TNFRSF5; 4, p53; 5, Fas/TNFRSF6/ CD95; 6, IκBε.

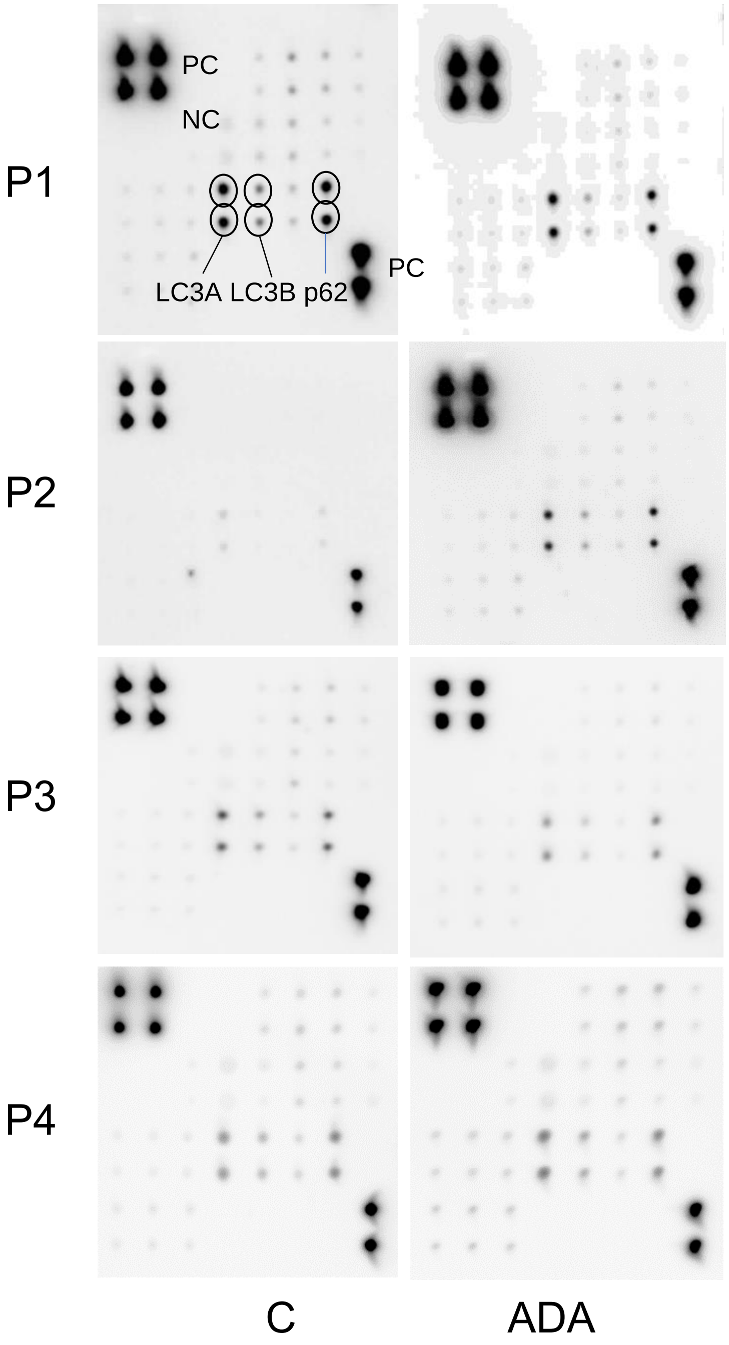

Figure S3. Comparative expression of the levels of the pro-apoptotic proteins LC3A, LC3B, and p62 in 3 patients with HS Hurley stage II (P1-P3) and one patient with HS Hurley III (P4) in HS lesional skin of the control group (C) and the adalimumab-treated group (ADA) maintained for 3 days in the HS 3D-SeboSkin model. PC = positive control, NC = negative control.
